# Institutional Medical Relief in London

**Published:** 1918-07-13

**Authors:** 


### INSTITUTIONAL MEDICAL RELIEF IN LONDON.

The Infirmary Medical Superintendents' Society at its annual meeting passed a resolution strongly approving the suggested transfer of the functions of Poor-Law authorities to the County, Borough and Town Councils, as outlined in the report of the Local Government Committee of the Ministry of Reconstruction, and decided to send the resolution to the Minister of Reconstruction, with a report showing the reforms the Society think desirable in the existing scheme of institutional medical relief in London as carried out under the Poor Law. This report is of great interest and importance as the carefully considered opinion of men who, as the administrative medical officers of large Poor-Law infirmaries, have, as regards both the Poor Law and medicine, the expert knowledge that enables them to point out authoritatively the defects of the present system and the reforms needed.

The Society is of opinion that the central administration of medical assistance over large areas would lead to economy; to the better distribution of patients amongst the institutions taken over; to the formation of medical and nursing services of such importance that they would attract better candidates than the present local services do; to uniformity in the training and certification of nursing probationers; and to the provision of institutions for special forms of treatment and of diagnosis, the cost of which in each of the present areas would not be justified, owing to the comparatively small number of patients who would benefit thereby. It suggests that London should be divided into convenient areas, and that in each area there should be a district hospital, with an out-patient department, and a Home for the aged and infirm. In addition there should be the following special institutions available for the whole of London: (1) A hospital equipped for the more elaborate forms of treatment and diagnosis by $x$-rays and electricity, for radium therapy, for pathological, bacteriological, and chemical investigations, for research work, and for testing drugs, foodstuffs, and other supplies; (2) special hospitals for the treatment of eye, ear, throat, and nose cases, for orthopædic cases, for dental treatment, and for chronic and advanced pulmonary tuberculosis, for such infectious diseases as measles, whooping-cough, and chicken-pox; (3) country and sea-side hospitals for curable cases of tuberculosis, for epilepsy, convalescent patients, etc. Central stores and ambulance service are recommended. As regards the administration of the medical work it is suggested that a principal medical officer should be appointed, and that he, with the specialist staff of the special hospitals and the medical superintendents of the district hospitals, should constitute a committee to advise the central authority on all questions of medical administration and assistance in the area; that the specialist staff should be available for consultation at the district hospitals, and that in each district the medical superintendent of the district hospital should be responsible for all the medical services organised by the County Council, other than those under the medical officer of health. We hope to deal on another occasion with the appendices attached to the report, which deal with the work of the district hospitals, the training of probationer nurses, and home medical assistance.

